# Genital Self-Mutilation in a Young Male With Psychotic Symptoms: Klingsor Syndrome – A Case Report

**DOI:** 10.1192/bjo.2025.10703

**Published:** 2025-06-20

**Authors:** Walaa Alnammourah, Nizar Marzouqa, Naim Alshaikh, Iltezam Morrar

**Affiliations:** Bethlehem Psychiatric Hospital, Bethlehem, Palestine

## Abstract

**Aims:** Genital self-mutilation (GSM) is a rare but severe form of self-harm often linked to underlying psychiatric disorders, particularly psychotic conditions. Approximately 54% of GSM cases occur in patients with psychosis, with substance use disorders being the second most common associated condition. Various triggers, including perceived rejection, lack of social support, and acute substance intoxication, have been implicated in GSM. When GSM arises from psychotic symptoms, it is referred to as Klingsor syndrome. Immediate psychiatric intervention is critical for managing such cases and preventing recurrence.

**Methods:** A 28-year-old divorced male was brought to the nearest hospital by his family following a penile self-amputation with a blade. Immediate surgical repair was performed. Three weeks later, he was admitted to Bethlehem Psychiatric Hospital for further evaluation and treatment. The patient had a history of self-harm that previously necessitated hospitalization. His psychiatric symptoms included commanding auditory hallucinations, delusions of reference, feelings of worthlessness, and psychotic features that emerged after cannabis use. In the weeks leading up to the self-mutilation, the patient exhibited insomnia, social withdrawal, and a growing preoccupation with self-castration. On examination, he appeared distressed, with an irritable affect and poor insight into his actions. He expressed a strong belief that his genitals were “the source of all problems” and reported suicidal ideations, stating he “needed to get rid of his penis or else would commit suicide”. He also exhibited persecutory delusions, delusions of guilt, control, and thought broadcasting. A comprehensive psychiatric assessment confirmed a diagnosis of Schizoaffective Disorder, exacerbated by substance use. He was admitted to the psychiatric ward following medical stabilization and was treated with quetiapine 600 mg/day, titrated as needed, and carbamazepine for mood stabilization. Supportive psychotherapy aimed at improving insight and addressing delusional distress was initiated, alongside family psychoeducation to prevent recurrence.

**Results:** During his four-week inpatient stay, the patient demonstrated gradual improvement in his psychotic symptoms. His insight improved significantly, and he ceased expressing delusional beliefs about his genitals. Upon discharge, he was referred to the Community Mental Health Centre (CMHC) and enrolled in an outpatient psychiatric programme, which included ongoing medication management and psychotherapy. At the few-months follow-up, he remained adherent to his treatment plan with no recurrence of self-harm behaviours.

**Conclusion:** This case highlights the interplay of psychosis and substance use in GSM and underscores the necessity for early intervention and psychiatric care. A multidisciplinary treatment approach including pharmacotherapy, psychotherapy, and family support is essential for prevention of future episodes.

